# A longitudinal study of the effect of individual and socio-cultural factors on students’ creativity

**DOI:** 10.3389/fpsyg.2023.1068554

**Published:** 2023-03-20

**Authors:** Hye-sook Park, Seokmin Kang, Sungyeun Kim

**Affiliations:** ^1^Graduate School of Education, Honam University, Kwangju, Republic of Korea; ^2^The Institute for Educational Research, Yonsei University, Seoul, Republic of Korea; ^3^College of Education and P-16 Integration, The University of Texas Rio Grande Valley, Edinburg, TX, United States; ^4^Graduate School of Education, Incheon National University, Incheon, Republic of Korea

**Keywords:** creativity, self-regulation, longitudinal study, socio-cultural influence, individual, statistics, statistics

## Abstract

This longitudinal study investigated how characteristics of individual and social relationships affect Korean students’ creativity development. Fifth graders (male: 3,623, female: 3,701) from 242 schools in Korea were followed annually from their 5th to 9th grades (indicating from the 5th elementary school grade to the 3rd middle school grade in the Korean school system). Exploratory factor analysis, internal consistency reliability (coefficient alpha), confirmatory factor analysis, and two-level growth model methods were performed. We investigated all nine constructs and their related items by checking metric and scalar invariance assumptions. When the measurement invariance assumptions were satisfied, we used the mean of items that constitute respective factors. We checked growth trajectories of creativity and tapped the possibility of the existence of subgroups based on the growth/change pattern using latent class growth modeling. The results showed that no subgroups existed. Thus, we constructed a two-level growth model to investigate the overall growth pattern of the students. Regarding level 1, we included time-varying variables such as peer attachment, self-regulation habits (self-management), parents’ academy-oriented involvement, parent affective support, individualized, interactive teaching methods, teachers’ academic pressure, and academic achievement. At level 2, we used gender and parenting style that was obtained at time point 1. The final combined model incorporating level 1 and 2 variables showed that students’ self-regulation had the most association with the student’s creativity followed by peer attachment, parents’ academic support, interaction with parents, interaction with teachers, academic pressure from teachers, and relationships with teachers. Methods for enhancing students’ creativity were discussed.

## 1. Introduction

Creativity refers to when one solves a problem in a way different from the existing methods or verifies usability by applying previous knowledge to a new area (Sternberg and Lubart, 1996). Beyond the personal dimension, various factors in the social context interact with each other and influence the development of creativity. Because of its novelty and usability, creativity has received considerable attention across various academic fields, including sociology, management, humanities, and education. Although the word “creativity” seems to encompass fancy features and has been defined in different ways, its core idea includes an ability to display originality, imagination, and expressiveness (American Psychological Association, 2023). Creativity is an individual’s ability to solve new problems with novelty by connecting various strategies and possibilities. Considering that creativity is an abstract concept, it is difficult to measure its construct. Thus far, creativity researchers have investigated it as a measurable ability and sought to discover its constituting factors. The measure is achieved sometimes through performance-based tasks such as by analyzing artifacts produced by an individual or by asking an individual to perform certain tasks (Antonietti and Colombo, 2012), and sometimes through self-report measures (Silvia et al., 2012). Importantly, self-report measures are usually not meant to serve as proxies for creative abilities or skills, but rather to reflect an individual’s beliefs about one’s own creativity. Efforts have been focused on understanding how a creative attitude is influenced by an individual’s cognition, attitude, and environmental factors (Csikszentmihalyi, 1999; Kim et al., 2002). In this study, we consider self-report, an individual’s own reflection of their attitude and behaviors, as a tool to measure creative ability. We address here the factors that we consider have influenced an individual’s creativity.

### 1.1. Cognitive factors

One approach to creativity research explores the cognitive characteristics of creative thinking, such as divergent (Guilford, 1950), associative (Mednick, 1962), analytical (Sternberg et al., 2005), and flexible thinking (Kenett et al., 2018). Researchers who view creativity from a cognitive perspective have focused on the possibility of it being an individual competence that can be measured through experimentation or a psychometric method. Guilford (1967) and Torrance (1980) proposed that creativity is related to cognitive abilities, especially divergent thinking skills, such as sensitivity to problems, fluency in thought, flexibility, originality, and elaborateness for segmenting and clarifying things. There is a rich body of research on the cognitive processes involved in creativity (Sawyer, 2012; Lee and Therriault, 2013; Cassotti et al., 2016). It is assumed that conflict monitoring (Ruzzoli et al., 2020), inhibitory control (Radel et al., 2015), and working memory (Beaty et al., 2014) are related to creative activities. Specifically, people’s ability to manage thinking processes through cognitive control and inhibition can be revealed through their behavior, which is then connected to how they control their thinking process when working on a certain task (Buckley et al., 2014; Tiego et al., 2018).

Considering the underlying cognitive mechanisms embedded in cognitive control, those engaged in creativity share many common features with those involved in self-regulation. According to Zimmerman (2001), “self-regulation refers to the self-directive process through which learners transform their mental abilities into task-related skills.” Managing one’s thinking and behaviors needs cognitive ability. It is the process of managing and monitoring one’s thinking and problem-solving processes to reach a designated goal (Berk, 2003). Specifically, self-regulated learning monitors and controls how students interact with learning tasks in their everyday lives (Zielińska et al., 2022a,b).

From the students’ perspective, self-regulation includes allocating adequate mental resources to an appropriate stage in the problem-solving or thinking processes (Shell et al., 2013). It is also known to mediate the transformation of creative ideas into creative products (Beeftink et al., 2012; Ivcevic and Nusbaum, 2017) and competency (Shell et al., 2013); moreover, it works collectively with metacognition to help creative thinking (Whitebread et al., 2009; Lizarraga and Baquedano, 2013). Considering that students’ creativity is evaluated in a school setting, measuring the students’ planning and controlling learning behaviors could be a strong predictor of creative behaviors and products (Rubenstein et al., 2018; Zielińska et al., 2021). We hypothesized that their self-regulation behaviors are positively related to their creativity.

Owing to its nonlinearity and multilayered characteristics, research on creativity needs to consider a broader but structured approach to understand its underlying nature. Aligned with its relationship to cognition, creativity is highly influenced not only by an individual’s competence but also by dynamics in an ecological system.

### 1.2. Social and ecological factors

According to Bronfenbrenner’s (1974) ecological framework, an individual interacts with and is influenced by various surrounding ecological systems. Especially for students in an education system, environmental factors, including the home ecological system and the school culture greatly impact children’s behavior, creative attitude, and thinking process, in addition to their innate qualities. As part of the home ecological system, parents’ attitudes are critical in forming a child’s creative characteristics (Pugsley and Acar, 2018). The former’s knowledge and beliefs about their parenting are related to their supportive behaviors toward their children, which in turn are related to the latter’s classroom behaviors (Bornstein et al., 2018). In addition, parents’ less supportive relationships with teachers implicate their children’s low achievement in school (Hughes and Kwok, 2007). A socio-cultural factor influences how individuals approach and solve a problem. For example, people from diverse cultures exhibit differences in their preferred creative processes (Rudowicz and Yue, 2002; Chua, 2018). Previous studies have indicated how micro-, meso-, and exo-systems and socio-cultural environments inevitably interact with each other influencing an individual’s development; this is revealed as students’ knowledge, attitudes, and behavior (Schilhab and Esbensen, 2019; Newman and Newman, 2020). At the same time, these interact with each other in all aspects of the students’ lives, including their knowledge, creative attitude, and behavior (Bronfenbrenner, 1986).

Through a multivariate behavioral genetic analysis, Kandler et al. (2016) found that creativity was explained more effectively by environmental factors. Again, this indicated that students’ creative behavior and attitude are closely related to their home and school environments, such as parenting style and interaction with parents, relationships with teachers, and school culture. Regarding the social factors’ influence on pupils, the home and school environments are inevitably mentioned. In this study, the home environment included parenting style, where a fosterer interacts with children, and the aspects they emphasize in everyday life. Hoferichter et al. (2021) found that German students from grade 7 and 8 who perceived their parents as supportive showed satisfaction with more various dimensions than those who perceived their teachers or peer as supportive.

Parents have the most impact on child development. Due to the significant influence of parenting styles on children’s cognitive, behavioral, and emotional development (Baumrind, 1966; Maccoby and Martin, 1983; Mehrinejad et al., 2015; Kuppens and Ceulemans, 2019; Pérez-Fuentes et al., 2019), parenting styles are the fundamental social factor influencing students’ creativity. Students’ creativity should be considered within the concept of parenting style and its relation to their creative attitude. Since Baumrind (1966) initially categorized three types of parenting styles, Maccoby and Martin (1983) expanded it using a two-dimensional framework that included authoritative, authoritarian, permissive, and uninvolved styles. The core idea is that children perceive the world and cope with problems differently, depending on the degree of parents’ responsiveness and demands. For example, an authoritarian style is based mainly on controlling a child, which is negatively related to creativity (Lim and Smith, 2008; Mehrinejad et al., 2015). However, authoritative and permissive styles are based chiefly on care and interest in the children, which was found to support creativity (Miller et al., 2012; Fearon et al., 2013; Popescu et al., 2015). An earlier review by Miller and Gerard (1979) demonstrated that the parents of creative children show respect, independence, and freedom toward their children. While the parents’ broad acceptance of their children’s performance outcomes is positively related to the latter’s creativity (Fan and Zhang, 2014), restraining them excessively, such as expecting higher grades or performance, diminishes it (Jankowska and Karwowski, 2014).

The key to the relationship between a parenting style and its impact on children is whether the style implemented by parents or nurturers is perceived by the children as intended. For instance, a father sets a clear rule to support his child’s behavioral needs, which is a feature of authoritative parenting. However, this can be interpreted as extreme pressure or a burden on the child. Thus, it can be considered an authoritarian style. In this sense, parenting style is to be judged from a child’s perspective.

Creativity relies on creative self-concept (Karwowski et al., 2019) that is greatly influenced by parent–child interactions. Therefore, parental support is a critical resource that can equip individuals with what they need; it simulates how things happen before stepping toward an unknown world. Parental support is closely associated with adolescents’ decision-making regarding their career choice (Kush and Cochran, 1993) and efficacy in pursuing mathematics and science subjects (Lopez et al., 1997). Therefore, parental cognitive and affective support function as stepping stones before and during the life-long journey; furthermore, their impact continues throughout an individual’s life (Neitzel and Stright, 2003; Leerkes et al., 2011). Accordingly, we hypothesized that the parents’ cognitive and affective support would be positively related to their children’s creativity.

A teacher’s pedagogical knowledge is another key aspect influencing students’ creativity; this is because it determines how the former interacts with the latter and what aspects are emphasized, which in turn influences the students’ ways of thinking, approaching a problem, and curiosity. Specifically, the student-teacher interaction patterns influence the level of pupils’ creative products (Pellegrini, 1984; Torrance and Goff, 1989; Kupers and van Dijk, 2020). It has been argued that educators effectively develop creativity and share common characteristics, some of which include providing an atmosphere of acceptance, asking thought-provoking questions, and valuing originality (Clark, 1983). Torrance and Goff (1989) suggested that teachers who provide students with opportunities to improve divergent problem-solving abilities will further enhance their development of creative thinking abilities. By analyzing student-teacher interaction patterns and products, Kupers and van Dijk (2020) investigated the process of how creativity emerges in the interaction between educators and students using a musical composition task. They found that convergent interaction between them created a barrier to creative output. This result aligned with the findings of other previous studies (Torrance and Goff, 1989; Lee and Kemple, 2014). For example, Torrance and Goff (1989) reported that the way teachers respond to students, such as creating a more responsive environment, is essential to the establishment of creativeness *via* the teacher-student relationship. The findings shared the idea that the teachers of young pupils were able to motivate them to demonstrate creative behaviors through positive verbal interactions (Feitelson and Ross, 1973; Pellegrini, 1984). Hence, we hypothesized that teacher support would be positively related to students’ creativity.

Peer attachment plays an important role in students’ lives. In particular, during adolescence, relationship with peers have a greater impact than those with teachers. For example, negative peer relationships such as bullying are correlated with low academic self-efficacy (Andreou and Metallidou, 2004). Peer attachment includes the concepts of respect, care, and trust, and encompasses cognitive and affective bonds.

Positive peer relationships, especially in adolescence, are related to the development of positive affect (Jose, 2015); moreover, they are helpful in overcoming negative family problems (Gauze et al., 1996; Hodges et al., 1999). They are also good predictors of academic self-concept (Shulman, 1993; Calero et al., 2014). Students’ high academic self-concept is positively associated with self-regulation strategies such as diligence, conception, and information processing (Ommundsen and Lemyre, 2007). These findings imply that peer attachment is influential and mediate many factors related to creativity. Although peer attachment is an important factor in both academic performance and affective stability, limited studies have explored the relationship between peer attachment and creativity, especially for students. We hypothesized that peer attachment would be related to creativity. Several attempts have been made to explore the complex dynamics embedded in creativity (Mumford and Hunter, 2005; Shell et al., 2013; Kharkhurin, 2014; Wu et al., 2014), studies have rarely investigated the longitudinal interrelationship of environmental factors and their impact on students’ creativity with a multi-level approach. Thus, accepting the integrative perspective of creativity (Amabile, 1983; Kim et al., 2002), this study aimed to discover the characteristics influencing students and their surrounding environmental aspects regarding their creative attitudes. Accordingly, we investigate how individual and social factors surrounding the students interact with each other and how these interactions influence their creativity.

We hypothesized that (1) individuals’ cognitive (self-regulation skills) and socio-cultural factors interact and affect their creativity, and (2) the sociocultural factors will have varying degrees of influence such as parenting style, peer factors, and interaction with teachers. The concept of creativity transcends a person’s cognitive aspect and reflects time and society; therefore, investigating how creativity is influenced by the society and culture that an individual learner belongs to has many implications in the sense that it can help society and schools mutually create enhanced learning environments for students to prepare for an uncertain future.

### 1.3. Gender

Considering the cognitive and socio-cultural influences mentioned above on creativity, this study considers gender that may moderate creative attitude and behaviors. Previous empirical studies have shown inconsistencies in gender superiority. Further, research targeting adolescents have demonstrated mixed results in the relationship between gender and creativity. This study examines how gender associates with creativity when both cognitive and sociocultural factors are considered. First, in some studies, males had higher levels of creativity (Zheng and Xiao, 1983; Rajendran and Krishnan, 1992; Abraham et al., 2013; He and Wong, 2021). For example, Zheng and Xiao (1983) compared male and female students’ creativity based on divergent thinking and creativity ratings by students’ teachers. They found that the former indicated higher creativity than the latter. Abraham et al. (2013), in their behavioral and functional magnetic resonance imaging research, showed that while there was no behavioral difference between males and females regarding various creative thinking tasks, only certain brain regions for both groups were activated during the tasks. In a divergent thinking exercise, males and females showed strong activation in brain areas related to declarative memory and social perceptions, which are central to the theory of mind and the theory of self-referential processing, respectively. This finding implied the involvement of a distinctive neurocognitive mechanism in creative thinking; men are more task-oriented, while women are more socially oriented.

Second, a series of studies reported higher creativity among female students (Kim and Michael, 1995; McCrae et al., 2002; Misra, 2003; He, 2018; Ivcevic et al., 2022). He (2018) explored a dynamic pattern of gender differences in creative thinking through 4 years of a longitudinal study, in which creative thinking for 775 participants from three age groups (children, adolescents, and emerging adults) was assessed using creative thinking drawing production and compared at one-year intervals. This study found that females had higher creativity during early adolescence.

Lastly, some studies indicated no gender differences (Kaufman, 2006; Baer and Kaufman, 2008; Cheung et al., 2010; Taylor and Barbot, 2021; Zielińska et al., 2022a,b). Although there was a gradual increase in the grade level, no gender difference was found between grades 1 and 9 (Cheung et al., 2010). In their review research, Baer and Kaufman (2008) showed that there were more studies with no gender differences in creativity than those with male or female superiority. Kaufman (2006) assessed the creative self-perceptions of 3,553 students and community members in 56 domains distributed across five factors. It was found that males scored higher on two of the five factors and 28 of the 56 domains, while females scored more on two factors and 15 domains. Considering the differences in the neural mechanisms’ dominant function (Abraham et al., 2013; Takeuchi et al., 2015), the gender gap relies on the creativity task, its involved cognitive and neural mechanisms, and the context in which the measure is administered. Since this study is based on students’ self-report of creativity in their problem-solving and divergent thinking behaviors and attitude in a school setting where it was found that the influence of peers is maximized, especially during adolescence, we hypothesized that at some points, the male students would be more creative than their female counterparts.

### 1.4. Age

**Figure 1:**
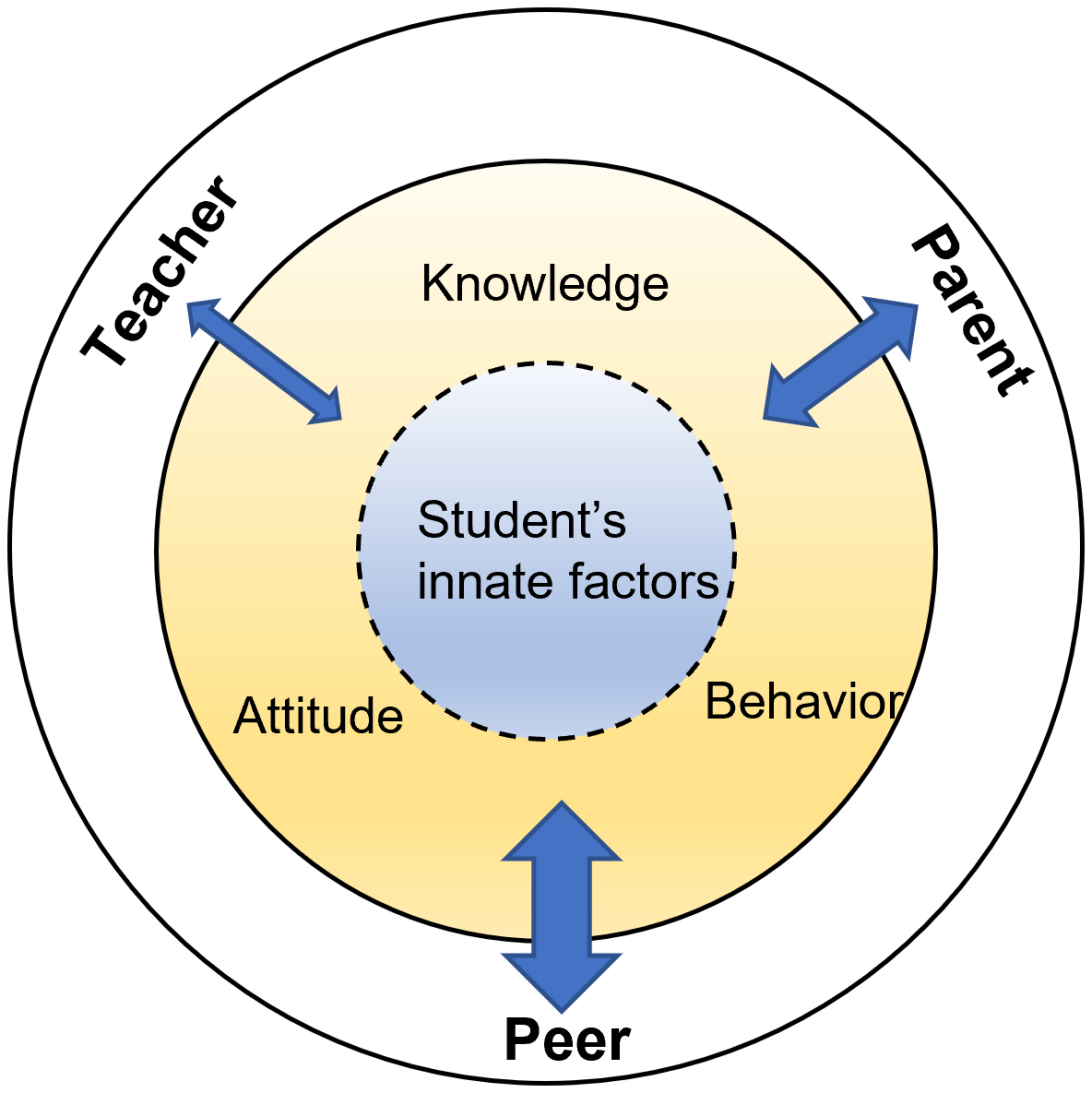
Observed influence of individual and social factors on a student’s creativity (Arrows indicate influential power).

The above-mentioned approaches need to be further considered with age-related variables (Sternberg, 1985; Razumnikova and Bakaev, 2022). This is because diverse cultural and contextual factors change constantly and influence individuals in distinct ways. Moreover, various social and ecological aspects are intertwined (Glǎveanu, 2010; Sawyer, 2012; Choe and Pyo, 2014; Glăveanu et al., 2020). This notion is represented in Figure 1 as a conceptual framework of the study. Choe and Pyo (2014) found that people in their twenties recognized creativity differently from those in their fifties. Given that the concept of creativity can be perceived and recognized differently over a period of time even within the same cultural boundary, it is necessary to conduct longitudinal studies of creativity across different time points. These findings highlight the necessity and importance of tracking changes in creativity over time. Similarly, Lubart (1999) suggested investigating changes among generations regarding the concept of creativity to better understand the influence of environmental context. The socio-cultural context’s impact on creativity has been supported by many previous studies that demonstrated creativity-related personality traits, including different socio-cultural factors (Miller and Gerard, 1979; Oral et al., 2007; Kaufman et al., 2009; Kandler et al., 2016). Although there are various ways of defining creativity, we used the term “creativity” as characteristics of association, flexibility, and uniqueness, given that we utilized the existing survey data which assessed creativity covering the three aspects mentioned above.

In this study we investigate from grade 5 to grade 9 (before high school year). Because the Korean Education Longitudinal Study (KELS) survey data was collected from 5th grade students (starting point), and full scaled preparation for college entrance exam starts from high school age in Korea, where majority of the students only focus on practicing exams to get higher scores by attending private institute or attending crash courses. We assumed that these learning behaviors during high school period do not adequately correspond to creativity. Specifically the followings are our research questions:

1. Whether and how the elementary students’ creativity changes across 5 years? (no change, linear trend or curved trend).
2. Whether and how are individuals’ cognitive (self-regulation skills, academic achievement) and socio-cultural factors (parents, peers, teachers) associated with the students’ creativity.
3. Whether and how do gender moderate the effect of the students’ other individual (self-regulation skills, academic achievement) and socio-cultural factors (parents, peers, teachers)?
4. Does growth trajectory differ based on gender?

## 2. Methods

### 2.1. Subjects

The subjects were from the Korean Education Longitudinal Study (KELS) 2013. Korean Educational Development Institute (KEDI) has been collecting data from 5th grade elementary students, their parents, teachers, principals, and schools from 2013 and it is available upon request through its home page. It was designed to collect students’ educational experiences, outcomes, school environment, educational activities, and organization climate longitudinally to obtain empirical evidence on the effect of educational policy, to assess educational outcomes, and to find out the results of social/class mobility through education. The survey was administered to those who consented *via* mail with the help of a professional organization with the approval of the board of IRB housed in the KEDI. Using a three-stage stratified sampling method, i.e., geographical region, school level, and class level across the whole South Korea, were collected. The study data are from the 5th graders obtained for 5 years (till 9th grade equivalent to Korean middle school 3rd year). Overall, 7,324 students participated in this survey. Among them, 3,623 (49.5%) and 3,701 (50.5%) were male and female students, respectively. The survey responses were followed annually from the 5th to the 9th grade, so there were some missing cases. In case of responses to creativity, only the completed cases were considered for analysis (see Table 1).

**Table 1:** Missing pattern.

Creativity	Frequency	Percent (%)	Completed cases
y1	49	0.7	7,077
y2	140	1.9	6,844
y3	512	7.0	6,310
y4	652	8.9	6,050
y5	812	11.1	5,786

In the case of year 1 (*r* = −0.045), year 3 (*r* = −0.026), and year 4 (*r* = −0.048), academic achievement was negatively correlated with those missingness, but the correlation size was negligible. In year 3, self-management was positively correlated with missingness (*r* = 0.024). In year 5, missingness was positively correlated with interaction with parents.

Based on our review of research, we hypothesed that (1) students’ creativity is not stable, it will be changing over 5 time points, (2) individuals’ cognitive and socio-cultural factors such as parents and home environment, friends, and teachers at school influence the students’ creativity, (3) some variability will exist in growth pattern (subgroups may exist or individual growth pattern may differ around mean).

### 2.2. The KELS child survey items

We identified groups of items related to creativity, self-regulation (self-management), peer attachment, parenting, teacher, and academic achievement which were recognized as individual constructs. The parenting style and support constructs comprised authoritative vs. autonomy-related parenting style, study-supportive parent assistance, and parent–child interactions. In addition, the perceptions of teachers/school culture, teaching methods (individualized and interactive instructions), and teachers’ interactions with students were identified.

The survey items were measured on a five-point Likert scale. The five items related to creativity were: (1) I can figure out things that many friends cannot; (2) I can think of things that can be helpful in solving new problems; (3) I can imagine the whole content even if I hear only a part of it; (4) I can make things connected each other even though they appear unrelated; and (5) I can find many new ideas in a short time. The students’ regulation-related items were also identified: (1) I plan things before undertaking whenever I have plenty to do; (2) I do not procrastinate on what I need to do today; (3) I check my planner/notice book and do not miss things that I need to do today; (4) I organize and clean my desk by myself; (5) I myself prepare/bring out things for school to work.

Regarding parenting, two groups of items were obtained at each time point and employed for level 1 predictors: (1) Eight items were related to the parent’s efforts to support their children’s education; and (2) Five items were associated with the parent’s relationship with their children. Two groups of items obtained at time point 1 (elementary fifth grade) were used at level 2 as level 1 intercept predictors. These two groups of items were related to parenting style (authoritarian and autonomy-supportive parenting styles).

Regarding the students’ perceptions of their teachers, eight items were related to student-centered teaching methods, namely: (1) individualized and (2) interactive. In addition, four items concerning the perceived teachers’ pressure on the students’ academic aspects, and interaction with students were identified. Each individual item for the designated construct was found to be a component of the respective factors; the means of these items were used as individual variables for the growth model. In addition, peer attachment construct also was used. Basic academic ability was gathered using three subjects: Korean, English, and basic Math ability levels from elementary 5th grade to middle school 3rd grade (equivalent to 9th grade in the USA). These three subjects are considered the most important courses in scholastic aptitude tests for college-bound exams in an academic performance-oriented Korean society. For comparison purposes across the grades, ability information was obtained using vertical scaling. Thus, it was possible to investigate the effect on creativity longitudinally by examining the rate of change in this variable. The mean of the three subjects was employed as a level-one predictor.

### 2.3. Methods of analysis

In this study, exploratory factor analyses were conducted to identify whether relevant items were one factor, and confirmatory factor analyses (CFA) were conducted to examine measurement invariance assumptions which were a prerequisite for longitudinal analysis of a measure. If measures do not have the same meanings across different time points, the study becomes invalid (Chen, 2007). We used Mplus 8.8 for CFA. Coefficient alpha was also obtained using SPSS 26 on the identified items that constitute one factor, respectively, each year.

Since the data were obtained over 5 years, we checked the possibility of utilizing the items constituting nine respective constructs (i.e., psychometric equivalence of each construct) across five time points; to see whether the constituting items are interpreted in the same way across five time points and the mean of each construct can be comparable across five time points. We checked metric and scalar invariance assumptions of the nine constructs, such as creativity, self-regulation, peer attachment, parent academic support, parental interaction with their children, teacher’s individualized instruction, teacher’s interactive teaching methods, teacher’s academic pressure, relationship with a teacher using Mplus 8.8 (see Table 2).

**Table 2:** Variables constructed and coefficient alpha.

Variable	Year	Alpha (*α*)	*N*	Content
Creativity	Y1	0.878	5	① I can figure out things that many friends cannot; ② I can think of things that can be helpful in solving new problems; ③ I can imagine the whole content even if I only hear only a part of it; ④ I can make things connected each other even though they appear unrelated; ⑤ I can find many new ideas in a short time
	Y2	0.893		
	Y3	0.898		
	Y4	0.900		
	Y5	0.912		
Management	Y1	0.773	4	① I plan things before undertaking whenever I have plenty to do; ② I do not procrastinate on what I need to do today; ③ I check my planner/notice book and do not miss things that I need to do today; ④ I organize and clean my desk by myself; ⑤ I myself prepare/bring out things for school to work.
	Y2	0.762		
	Y3	0.754		
	Y4	0.765		
	Y5	0.773		
Peer-attach	Y1	0.914	6	Friendship relationship - peer attachment: ① My friends respect my thoughts when talking with me; ② My friends listen to what I say; ③ I tell my friends about my worries and problems; ④My friends understand me well; ⑤ I can tell my friends when I want to confide in my heart; ⑥ I trust my friends.
	Y2	0.909		
	Y3	0.917		
	Y4	0.929		
	Y5	0.930		
P_Ac_sup	Y1	0.806	6	① Parents create an environment for study at home; ② Parents check school studies and homework; ③ Parents give advice on my study; ④ Parents participate in my grade management; ⑤ Parents collect information to determine tutoring or private institutes; ⑥ Parents check my daily work/routine and manage my schedule.
	Y2	0.802		
	Y3	0.867		
	Y4	0.867		
	Y5	0.861		
P_Interaction	Y1	0.786	4	① My parents are concerned about me and ask questions regarding my school life; ② They respond to my questions kindly no matter how tedious they are; ③ They play games with me; ④ They read books together and talk.
	Y2	0.803		
	Y3	0.840		
	Y4	0.834		
	Y5	0.833		
T_individualized	Y1	0.890	4	① Teachers are well aware of my strengths and weaknesses; ② Teachers check my level of understanding; ③ Teachers give homework according to my abilities; ④ Teachers explain according to my level.
	Y2	0.902		
	Y3	0.898		
	Y4	0.912		
	Y5	0.914		
T_Interactive	Y1	0.853	5	⑤ Teachers frequently praise me as a means to encourage me to study more; ⑥ Teachers encourage me to challenge even a slightly difficult problem; ⑦ Teachers give me ample opportunities to present myself in class; ⑧ When I ask questions that I do not understand, my teachers kindly explain things again until I understand.
	Y2	0.904		
	Y3	0.885		
	Y4	0.890		
	Y5	0.895		
T_Pressure	Y1	0.718	4	② Teachers dislike students if they do not study hard; ③ My teacher emphasizes that our class should excel in the tests; ④ My teacher emphasizes that all students should complete their homework; ⑤ My teacher thoroughly inspects my homework.
	Y2	0.710		
	Y3	0.714		
	Y4	0.706		
	Y5	0.730		
T_relations	Y1	0.925	5	Relationship with teacher – ① He/She listens to me well; ② My teacher calls my name kindly; ③ When I say hello, he/she is welcoming; ④ My teacher often praises me; ⑤ My teacher knows me well.
	Y2	0.925		
	Y3	0.905		
	Y4	0.916		
	Y5	0.923		
P_authoritarian	Y1	0.712	2	① Parents ordered me to follow their words/what they say; ② Parents interfere with my work/small things.
P_autonomy	Y1	0.772	2	① Parents respect my decision; ② Parents allow me to choose what I want to do.

Management, Peer_attach, P_Ac_sup, P_interaction, T_individual, T_interaction, T_Pressure, and T_relations represent self-management, peer attachment, parental academy-supportive learning environment, parent–child interactions, teachers’ individualized teaching methods, interactive teaching methods, teachers’ academic pressure, and children’s relationships with teachers, respectively. The circled numbers are the item ids given by the KELS2013. P_authoritarian and P_autonomy represent authoritarian and autonomy-supportive parenting styles, respectively; these two variables are used for Level 2.

When the metric invariance assumptions were satisfied at least, we obtained the means of the items that constitute each construct at each time point and used them as respective constructed variables. Then we checked how and whether the students’ creativity changed over five time points and whether there existed any subgroups in the growth trajectory (growth pattern). If there existed any subgroups, we wanted to employ a growth mixture modeling to find out which variables contributed to the membership of the possible subgroups. When we found out that there were no salient subgroups (a group with more than 5 percent of students), we regarded the sample as one group (Jung and Wickrama, 2008).

**Figure 2:**
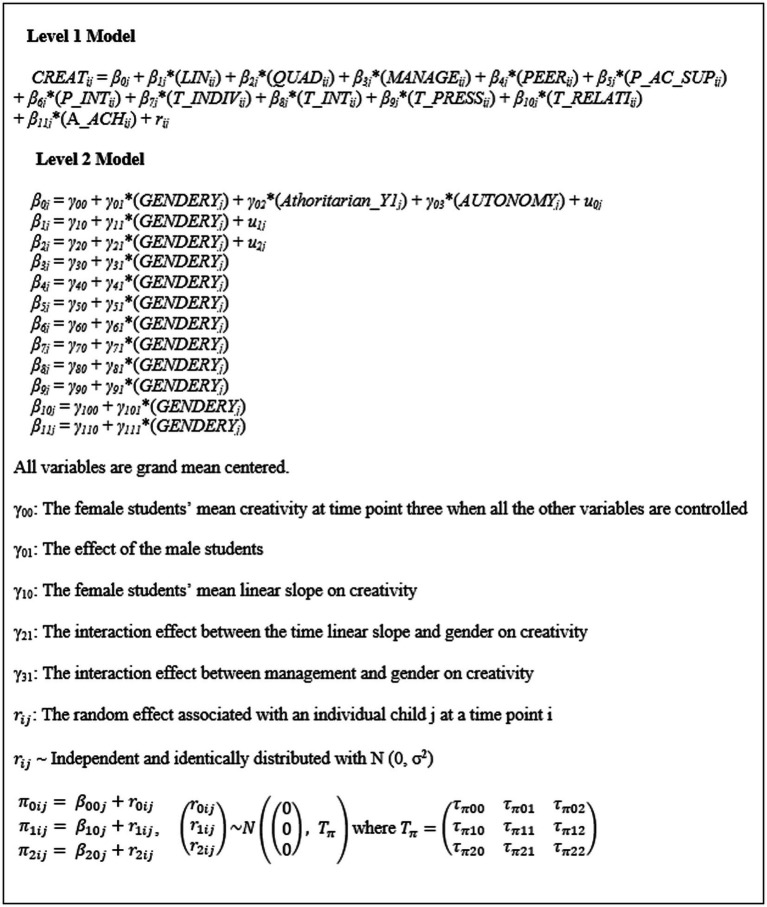
Final model: a two-level growth model.

A two-level growth modeling was employed to assess the overall growth trajectory and the effect of each constructed variable on creativity. In the two-level growth modeling analysis, time-varying characteristics (variables) were nested within the individuals. Level one coefficients are predicted by level two variables. We incorporated the nine constructed covariates such as creativity, self-regulation (self-management), peer attachment, parental academy-supportive learning environment, parent–child interactions, teachers’ individualized teaching methods, interactive teaching methods, teachers’ academic pressure, and children’s relationships with teachers, and academic achievement that were obtained at each time point at level 1. Gender and two other constructed variables such as authoritarian and autonomy-supportive parenting styles that were obtained at time point 1 were incorporated at level 2 as level 1 intercept predictors. In addition, we also incorporated gender for level 1 slope predictor (including linear and quadratic slope) at level 2. Figure 2 represents our two level growth model.

## 3. Results

To tab the feasibility of utilizing relevant items for their respective construct, we conducted a principal axis exploratory factor analysis with a direct oblimin rotation using SPSS 26. We first identified items that constitute the respective construct and also obtained internal consistency reliability using the same items across five time points. The items of each construct showed good reliability (see also Table 2). Table 3 showed descriptive statistics of the outcome variable, creativity. The items that constituted individual constructs turned out to be one factor; moreover, the constituting items for respective factors satisfied measurement invariance assumptions. Thus, the mean of the items constituting each factor was used as an individual variable.

**Table 3:** Descriptive statistics.

Item	*N*	Mini	Max	Mean	SD	Skewness	Kurtosis
Create_Y1	7,275	1	5	3.501	0.776	−0.083	−0.028
Create_Y2	7,184	1	5	3.633	0.759	−0.065	−0.131
Create_Y3	6,812	1	5	3.628	0.741	−0.029	0.007
Create_Y4	6,672	1	5	3.544	0.747	0.017	0.050
Create_Y5	6,512	1	5	3.591	0.766	−0.047	0.013

Y stands for Year. For example, Y1 means Year 1.

Before we investigated whether and how creativity changes over time, we checked both metric and scalar invariance assumptions of creativity along with other variables used for the final model. Creativity satisfied both metric and scalar invariance assumptions, and the other variables satisfied at least metric invariance assumptions. Since chi-square test was sensitive to sample size (Chen, 2007), we used other fit indices such as RMSEA, CFI, and TLI. If metric invariance model fit is not deteriorated compared to configural invariance model fit, metric invariance assumptions are considered satisfied. If the fit value of the scalar invariance model does not deteriorate compared to metric invariance model, the scalar invariance assumption is considered to be met (Cheung and Rensvold, 2002). Table 4 shows that all constructed variables used for the level 1 model satisfied at least metric invariance assumptions. Thus, we obtained the mean of the items that constitute each construct at each time point and used them as respective constructed variables and the nine variables were constructed.

**Table 4:** Configural, metric, scalar invariance assumption.

Variable	Model	*χ* ^2^	df	RMSEA (CI)	CFI	TLI
Creativity	Configural	2085.232	25	0.109 (0.105 ~ 0.113)	0.979	0.959
	Metric	2106.434	41	0.085 (0.082 ~ 0.089)	0.979	0.975
	Scalar	2267.717	57	0.075 (0.072 ~ 0.078)	0.978	0.980
Management	Configural	1529.285	10	0.148 (0.142–0.155)	0.959	0.877
	Metric	1590.915	22	0.102 (0.0097 ~ 0.106)	0.958	0.942
	Scalar	3499.311	34	0.122 (0.118 ~ 0.125)	0.906	0.917
Peer_attach	Configural	15564.16	45	0.224 (0.221 ~ 0.227)	0.91	0.849
	Metric	15694.71	65	0.187 (0.184 ~ 0.189)	0.909	0.895
	Scalar	16160.84	85	0.166 (0.163 ~ 0.168)	0.906	0.917
P_ac_sup	Configural	3991.521	45	0.113 (0.110 ~ 0.116)	0.952	0.920
	Metric	4091.983	65	0.095 (0.092 ~ 0.097)	0.951	0.944
	Scalar	7043.098	85	0.109 (0.107 ~ 0.111)	0.916	0.926
P_interaction	Configural	6283.426	10	0.301 (0.295 ~ 0.308)	0.906	0.719
	Metric	6360.156	22	0.204 (0.200 ~ 0.209)	0.905	0.871
	Scalar	8837.869	34	0.194 (0.190 ~ 0.197)	0.868	0.884
T_ind_instruction	Configural	4106.391	10	0.244 (0.237 ~ 0.250)	0.957	0.870
	Metric	4145.398	22	0.165 (0.161 ~ 0.169)	0.957	0.941
	Scalar	6054.071	34	0.160 (0.157 ~ 0.164)	0.936	0.944
T_interactive	Configural	3068.518	10	0.211 (0.204 ~ 0.217)	0.966	0.898
	Metric	3199.122	22	0.145 (0.140 ~ 0.149)	0.965	0.952
	Scalar	3449.533	34	0.121 (0.117 ~ 0.124)	0.962	0.967
T_pressure	Configural	5165.678	25	0.173 (0.169 ~ 0.177)	0.864	0.727
	Metric	5256.686	41	0.136 (0.133 ~ 0.139)	0.862	0.831
	Scalar	6338.478	57	0.126 (0.124 ~ 0.129)	0.833	0.854
T_Relation	Configural	3650.989	25	0.146 (0.141 ~ 0.149)	0.972	0.943
	Metric	3747.382	41	0.114 (0.111 ~ 0.118)	0.971	0.965
	Scalar	4878.001	57	0.111 (0.108 ~ 0.113)	0.962	0.967

We checked the growth trajectory of students’ creativity and found that a model with both linear and quadratic terms (variables) was better than the model with linear term alone, which means that the students’ creativity changed but not in a consistent pattern over 5 years (see also Table 5). A simple growth model demonstrated that a model with both linear and quadratic slopes (*χ*^2^ = 373.365, df = 6, CFI = 0.996, TLI = 0.994, RMSEA = 0.042) was a better fit than that with a linear slope only (*χ*^2^ = 1614.708, df = 10, CFI = 0.984, TLI = 0.984, RMSEA = 0.068), as indicated by the descriptive statistics of creativity (Table 3).

**Table 5:** Growth trajectory.

Model	*χ* ^2^	df	RMSEA (CI)	CFI	TLI
Lin	1614.708	10	0.068 (0.065 ~ 0.070)	0.984	0.984
Lin & Quad	373.365	6	0.042 (0.039 ~ 0.046)	0.996	0.994

We also tapped the possibility of the existence of any subgroups based on the growth/change pattern using Mplus. We found out that there did not exist any subgroups. Given the pattern of change, latent class modeling (LCGA) was administered. In order to be considered as a group, the smallest subclass/group was composed of more than 5% of the participants (Jung and Wickrama, 2008; see Table 6). Since less than 1 % of students were found in one of the classes/groups, we decided not to investigate the subgroups further. Instead, we investigated the general (average) growth trajectory of the whole sample using a two-level hierarchical linear modeling (HLM) because it assesses the random effects of each slope efficiently.

**Table 6:** Latent class growth analysis (LCGA).

N Class		Class (%)			Akaike (AIC)	Bayesian (BIC)	Entropy
2	0.004	0.996	__	__	366117.9	366269.9	0.998
3	0.979	0.004	0.017	__	364837.4	365023.3	0.974
4	0.003	0.874	0.056	0.067	363685.9	363905.5	0.860

The unconstrained model in a two-level growth model without any predictors showed that the intraclass correlation was about 0.47, indicating that approximately 47(=0.313/(0.313 + 0.356))% of the total variance was at level 2, within an individual’s stable aspects and the rest in the level 1 (53%; Table 7).

**Table 7:** Unconstrained model.

Fixed effect	*b*	*SE*	*t*	*df*	*p*
INTRCPT2, *γ_00_*	3.580	0.007	481.698	7,103	<0.001
Random effect	SD	Variance	df	χ^2^	*p*
INTRCPT1, *u_0_*	0.560	0.313	7,103	36485.97	<0.001
level-1, *r*	0.596	0.356			

**Figure 3:**
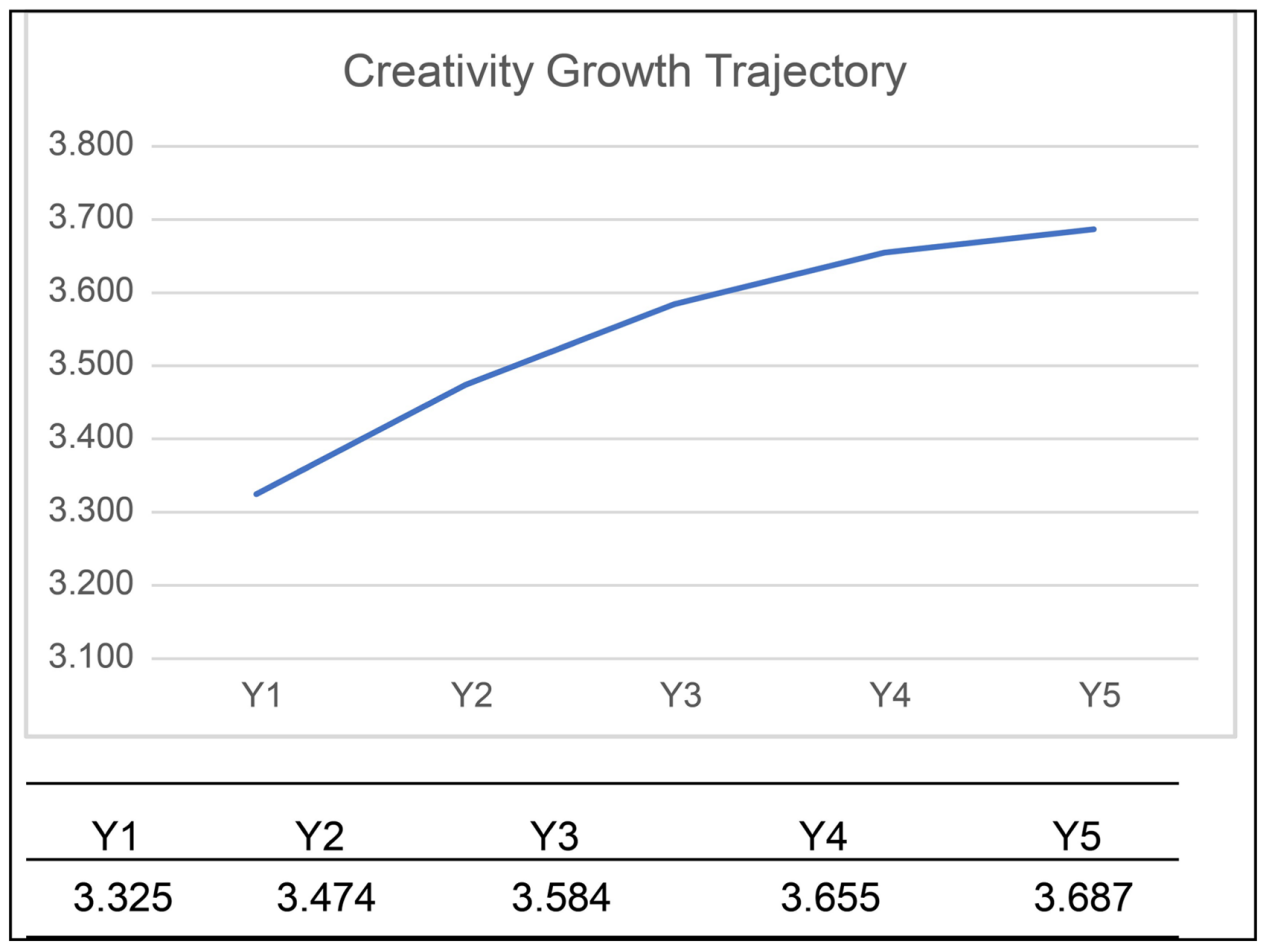
Growth trajectory of creativity of the students from the fifth to ninth grades.

We also found that the students’ creativity did not develop at a consistent rate but instead showed a nonlinear pattern (Figure 3). The quadratic effect was not extremely strong (the *t*-value was similar to that of the linear term). We did not include cubic terms in this study because no degrees of freedom were left with only five time-point data points (Table 8).

**Table 8:** Growth over time.

Fixed effect	*B*	*SE*	*t*	*df*	*p*
INTRCPT2, *γ_00_*	3.584	0.007	484.717	7,237	<0.001
INTRCPT2, *γ_10_*	0.091	0.008	11.13	7,237	<0.001
INTRCPT2, *γ_20_*	−0.020	0.002	−10.23	7,237	<0.001
Random effect	SD	Variance	df	*χ* ^2^	*p*
INTRCPT1, *u_0_*	0.569	0.324	6,761	39902.16	<0.001
LIN slope, *u_1_*	0.262	0.069	6,761	7933.016	<0.001
QUAD slope, *u_2_*	0.050	0.003	6,761	7510.049	<0.001
Level-1, *r*	0.550	0.302			

We ran a two-level growth model with changing variables for level 1, and included gender and the children’s perceived evaluation of their parents’ parenting styles (authoritarian vs. autonomy-supportive parenting styles) obtained at time point 1 for level 2. We grand mean-centered all the variables except gender to avoid the multicollinearity problem. If only linear term is used at level 1 of the basic growth model, then the intercept means the students’ mean creativity at grade 5 (the first time point when each time point is coded as 0, 1, 2, 3, 4). Since the predictors are all grand mean-centered which is the mean of all the time point data (7th grade), the intercept 3.581 indicated the average creativity at time point 3 (middle school 1st year, equivalent to grade 7 in the USA). When only linear and quadratic variables were employed, they were statistically significant. The intercept and slopes also varied randomly (*p* < 0.001), indicating that there were some differences in the mean creativity and individual differences in the growth rate. All predictors were positively associated with the children’s creativity (Table 9).

**Table 9:** A model with time-varying predictors only.

Fixed effect	*b*	*SE*	*t*	*df*	*p*
Intercept	3.580	0.006	588.072	7,103	<0.001
Lin, *γ_10_*	0.091	0.008	11.695	7,103	<0.001
Quad, *γ_20_*	−0.022	0.002	−11.845	18,790	<0.001
Managment *γ_30_*	0.209	0.007	31.892	18,790	<0.001
Peer_Attach, *γ_40_*	0.131	0.006	20.551	18,790	<0.001
P_Ac_Support, *γ_50_*	0.058	0.006	10.319	18,790	<0.001
P_Interaction, *γ_60_*	0.063	0.006	11.31	18,790	<0.001
T_Individualized, *γ_70_*	0.068	0.007	9.803	18,790	<0.001
T_Interative, *γ_80_*	0.037	0.007	5.391	18,790	<0.001
T_Pressure, *γ_90_*	0.044	0.005	8.637	18,790	<0.001
Rel_Teacher, *γ_100_*	0.023	0.007	3.349	18,790	<0.001
Acad_achievement, *γ_110_*	0.001	0.000	11.667	18,790	<0.001
Random effect	SD	Variance	df	*χ* ^2^	*p*
INTRCPT1, *u_0_*	0.444	0.197	6,983	29122.45	<0.001
LIN slope, *u_1_*	0.103	0.011	6,983	9413.125	<0.001
level-1, *r*	0.533	0.284			

Table 10 shows our final target model with both level 1 and level 2 predictors. Here, the intercept (*γ*_00_ = 3.586) indicates the average female children’s creativity at time point 3 when the remaining variables in the model were controlled. The effect of gender on the mean of the student’s creativity was statistically significant. Although the creativity of males was higher than that of females, gender did not have any statistically significant effect on the growth rate. However, gender was positively associated with self-regulation (*γ*_31_ = 0.051, *β* = 0.031) and peer attachment (*γ*_41_ = 0.037, *β* = 0.023), the effects of which were higher in males than in females. The effect of autonomy-supportive parenting style (*γ*_03_ = 0.056, *β* = 0.061) was positively related to the children’s creativity; nevertheless, the effect of authoritarian parenting was not statistically significant. Among all predictors, the effect of self-management (self-regulation-related construct) was the largest (*γ*_30_ = 0.208, *β* = 0.201). Even after incorporating the other variables in the model, the random effects of intercept, linear slope, and quadratic slope were statistically significant, indicating that there were individual differences in the mean and the growth rate.

**Table 10:** Final model: fixed and random effect.

Fixed effect	*b*	*SE*	*t*	*df*	*p*	*β*
INTRCPT2, *γ_00_*	3.586	0.006	596.19	7,234	<0.001	
Gender(M), *γ_01_*	0.167	0.012	13.798	7,234	<0.001	0.102
Authoritarian, *γ_02_*	0.012	0.006	1.889	7,234	0.059	0.014
Autonomy, *γ_03_*	0.056	0.007	7.996	7,234	<0.001	0.061
LIN, *γ_10_*	0.087	0.008	11.239	7,236	<0.001	0.151
Gender(M), *γ_11_*	−0.021	0.016	−1.32	7,236	0.187	−0.013
QUAD, *γ_20_*	−0.022	0.002	−12.032	7,236	<0.001	−0.156
Gender(M), *γ_21_*	0.006	0.004	1.734	7,236	0.083	0.004
Management, *γ_30_*	0.208	0.005	39.124	11,758	<0.001	0.201
Gender(M), *γ_31_*	0.051	0.011	4.77	11,758	<0.001	0.031
Peer_attach, *γ_40_*	0.135	0.005	24.903	11,758	<0.001	0.132
Gender(M), *γ_41_*	0.037	0.011	3.43	11,758	<0.001	0.023
P_Acad_Support, *γ_50_*	0.053	0.005	10.56	11,758	<0.001	0.058
Gender(M), *γ_51_*	−0.004	0.010	−0.392	11,758	0.695	−0.002
P_INT, *γ_60_*	0.059	0.005	11.991	11,758	<0.001	0.065
Gender(M), *γ_61_*	−0.009	0.010	−0.958	11,758	0.338	−0.006
T_INDIV, *γ_70_*	0.063	0.006	10.18	11,758	<0.001	0.067
Gender(M), *γ_71_*	−0.001	0.012	−0.07	11,758	0.944	−0.001
T_INT slope, *γ_80_*	0.036	0.006	5.715	11,758	<0.001	0.039
Gender(M), *γ_81_*	0.001	0.012	0.099	11,758	0.921	0.001
T_PRESS, *γ_90_*	0.040	0.005	8.725	11,758	<0.001	0.041
Gender(M), *γ_91_*	−0.010	0.009	−1.072	11,758	0.284	−0.006
T_RELATI, *γ_100_*	0.022	0.006	3.515	11,758	<0.001	0.022
Gender(M), *γ_101_*	−0.012	0.012	−0.985	11,758	0.324	−0.007
A_ACh, *γ_110_*	0.002	0.000	13.636	11,758	<0.001	0.090
Gender(M), *γ_111_*	0.000	0.000	−1.316	11,758	0.188	0.000
Random effect	SD	Variance	df	*χ* ^2^	*p*	
INTRCPT1, *u_0_*	0.436	0.190	6,758	28240.05	<0.001	
LIN slope, *u_1_*	0.208	0.043	6,760	7522.171	<0.001	
QUAD slope, *u_2_*	0.038	0.001	6,760	7208.738	<0.001	
Level-1, *r*	0.526	0.277				

LIN, Linear effect; QUAD, Quadratic effect.

**Figure 4:**
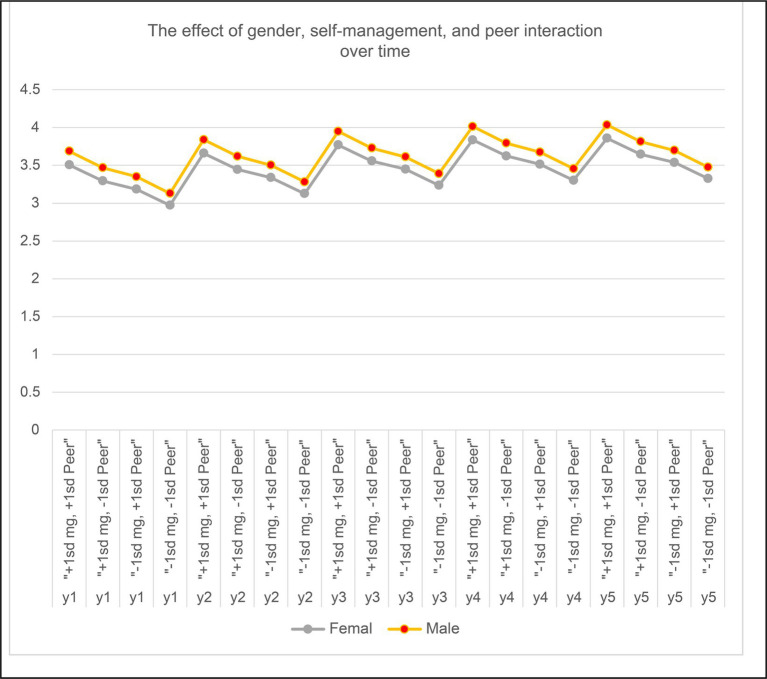
Growth trajectory of self-regulation and peer attachment.

Since both self-regulation and peer attachment were highly associated with creativity, we investigated the effect of these two variables along with gender. The effect of one standard deviation above and below the mean of the two variables (self-regulation and peer attachment) for male and female over the five time points has been displayed in Figure 4. The effect of one standard deviation above the mean of each variable demonstrated the highest creativity in the fifth year, followed by one standard deviation above self-regulation and one standard deviation below in peer attachment, respectively. The influence of gender on creativity was the largest at time point 5 (Figure 4; Table 11).

**Table 11:** The effect of self-regulation, peer attachment, and gender on creativity.

Year	Level of management and peer	Female	Male
y1	“+1SD” mg, “+1SD” Peer	3.505	3.688
y1	“+1SD” mg, “−1SD” Peer	3.293	3.467
y1	“−1SD” mg, “+1SD” Peer	3.184	3.350
y1	“−1SD” mg, “−1SD” Peer	2.972	3.130
y2	“+1SD” mg, “+1SD” Peer	3.659	3.840
y2	“+1SD” mg, “−1SD” Peer	3.447	3.620
y2	“−1SD” mg, “+1SD” Peer	3.338	3.503
y2	“−1SD” mg, “−1SD” Peer	3.126	3.282
y3	“+1SD” mg, “+1SD” Peer	3.769	3.949
y3	“+1SD” mg, “−1SD” Peer	3.557	3.728
y3	“−1SD” mg, “+1SD” Peer	3.448	3.611
y3	“−1SD” mg, “−1SD” Peer	3.236	3.391
y4	“+1SD” mg, “+1SD” Peer	3.836	4.013
y4	“+1SD” mg, “−1SD” Peer	3.623	3.793
y4	“−1SD” mg, “+1SD” Peer	3.515	3.676
y4	“−1SD” mg, “−1SD” Peer	3.303	3.455
y5	“+1SD” mg, “+1SD” Peer	3.859	4.034
y5	“+1SD” mg, “−1SD” Peer	3.646	3.814
y5	“−1SD” mg, “+1SD” Peer	3.538	3.696
y5	“−1SD” mg, “−1SD” Peer	3.325	3.476

+1 SD above mean of the management. −1 SD below mean of the management. +1 SD above mean of the peer attachment. −1 SD below mean of the peer attachment.

## 4. Summary and discussion

We focused on the change in creativity across five time points (5th grade to 9th grade) employing rigorous methods. For predicting creativity over time, measurement invariance assumptions were checked including all the constructed variables used for the growth modeling. In addition, to tap the possibility of subgroups based on the pattern of change, latent class modeling (LCGA) was conducted. The results of the LCGA showed no indication of subgroups (classes), therefore, we assessed the general (average) growth patterns employing a two-level hierarchical linear model. The children’s creativity did not change at a consistent rate, showing a curved trajectory. When only time-varying variables were used as level-one predictors, the variables selected were all significantly associated with creativity, where self-regulation as was reflected in self-management was most highly related to it, followed by peer attachment, teachers’ individualized instruction, and interactive teaching methods. Our target model with level 2 variables showed that the effect of gender on the mean of the student’s creativity was statistically significant, where the males’ level of creativity was higher than that of the females. However, in the growth rate, gender was not significantly associated with the change. Although there were no subgroups in growth trajectories, there existed individual differences around the mean growth trajectory and rate of growth as was reflected in the statistically significant random effects.

Additionally, we examined the interaction effect of all covariates and gender. The latter was positively associated with self-regulation and peer attachment. The effects of self-regulation and peer attachment were greater in male than in female students. While the impact of autonomy-supportive parenting style was positively related to the children’s creativity, that of the authoritarian parenting style was not.

The findings imply that creativity is not a product of an individual pupil only; instead, it is co-constructed through dynamic interactions with people around them. At level 1, self-regulation skills, peer attachment, parents’ academic support (cognitive support), interaction with parents (affective support), teacher’s individualized support (cognitive support), teacher interaction (affective support), teacher’s academic pressure, and relationship with the teacher significantly explained the students’ creativity. At level 2, both gender, parenting style, and autonomy-supporting parenting styles significantly explained creativity.

The male students showed higher creativity than their female counterparts. A gender effect was observed because children are socialized differently based on gender. Early research focused on gifted girls who primarily hide their giftedness, beginning in the pre-teen years (Silverman and Miller, 2009). It can be interpreted that girls are more sensitive to and conscious of peer reputations in their peer groups. This becomes more obvious during the early teenage period. This is related to the concept of talented profiles (Betts and Neihart, 1988). According to the profiles, female adolescents are more likely to hide or deny their talent in order to feel a sense of belonging to their peer group, which is called “underground.” The “underground” characteristics could have influenced the female students’ attitudes and behaviors, which could be fully expressive as creative thinking and behaviors.

Another interpretation of our finding regarding male superiority in creativity could be cultural influences, such as a male-dominant society and cultural stereotypes (Kaufman, 2006; Eisler et al., 2016; Shao et al., 2019) because social influences on gender may vary according to culture. With the rapid economic development and social reform, there are fewer restrictions in Korea in terms of women’s career selection and exploring their domains of interest. However, the latent consciousness deeply rooted in Confucianism remains relatively delayed regarding the rapid social and institutional change (Kim, 2009). For example, male students are more likely to be encouraged to explore the world than female students (Chaplin and Aldao, 2013). The female students in the study who are still under the custody of previous generations are more likely to be influenced by the previous generations’ thoughts and regulations. This collective, implicit force still imposes female students to stay quiet, inactive, obedient, not risk-taking, and withhold what is inside, not expressing it (Swim et al., 2010; Chaplin and Aldao, 2013).

We found that the students’ creativity developed nonlinearly. During elementary school, it showed a gradual increase, with the highest level at grade 6; subsequently, it decreased, being the lowest at grade 8 (middle school sophomore), and then slightly increased. Although there are some longitudinal studies investigating change in creative self-concept (Karwowski, 2015) and reciprocal relationships between creative self-efficacy and creative self-identity (Karwowski, 2016), We found no longitudinal studies that showed nonlinear pattern in creativity development of 5th graders over five time points. Although our creativity measure is different from studies with performance-based measure, there is correspondence in its nonlinearity (Cheung et al., 2010), where the students’ creativity (from grade 1 to 9) was measured using the Wallach-Kogan Creativity test; there was a general increase in the creativity score as the grade level increased, except that there was a significant drop in grade 7 for the figure tests regardless of gender. In this sense, investigating the relationship between self-reported creativity and performance-based creativity measure would be the next direction of our research. Once students enter their teenage years, their creativity ability seems to be regulated by social influence, especially peer relations, because the personal creativity potential begins to change during this period. Also, the interpretation aligns with our findings, where the peer influence on the students was greater than that on the parents and teachers. In affective aspects, quite a few studies found that, as children grow older, the effect of parents on creativity got reduced, compared to that of their peers (Montemayor, 1984; Smith, 2016). Considering that the students spent most of their time in school interacting with peers, the effect of peer influence could be large, which would have affected how they thought and behaved.

A parenting style that respects a child’s autonomy is significantly associated with creativity. Among the parenting styles, autonomy had a greater overall impact on the students’ creativity. This result is consistent with those of the previous studies (Fearon et al., 2013; Fan and Zhang, 2014; Mehrinejad et al., 2015). The common finding has been that parents’ acceptance of their children’s behaviors has a positive correlation with the latter’s creativity; however, the acceptance should be combined with parents’ care and interest in their children (Miller and Gerard, 1979; Popescu et al., 2015). On the contrary, authoritarian parenting did not explain the students’ creativity. We conjecture that creativity develops in a more comfortable environment to explore, but not in a rigid environment. All time-varying variables at level 1 significantly influenced creativity. Among these variables, competence in self-regulation as reflected in our model had a significant impact on creativity. Parental support, positive peer relations, and students’ academic performance-driven behaviors were closely related to creativity. Since a person’s innate and socio-cultural factors work collectively, an individual needs to actively engage in interacting with environmental factors to maximize creativity. This idea is represented in Figure 1, based on our analyses’ findings. Creativity is more likely to emerge when a student’s innate factors strongly which are influenced by peers. The relationship with the peers also simultaneously moderates the relationship between the parent and teacher aspects.

This finding corresponds to the study by Llorca et al. (2017), who found that peer relationships were mediated by parenting style and academic performance. In addition, we regarded teachers’ influence as part of school culture. Considering the unique performance-oriented school culture in Korea, if students in two different countries are compared, the teacher-related factors would show a slightly different pattern in their relationship with other variables and creativity. Although it was out of the scope of this study to find a causal relationship among parental styles, peer attachment, and creativity, it appeared that the former two variables closely worked together for the students’ regulatory behavior, thus influencing their creativity.

Overall, we found that creativity is not only based on both cognitive and affective support at both the individual and socio-cultural levels but also on how the various factors surrounding an individual influence it throughout adolescence, with the highest impact being peer attachment factors, followed by that of the parents and teachers.

This study still has some limitations. Considering that creativity is a multidimensional construct, including divergence, association, analytic ways, flexibility, uniqueness, and usability-related aspects, creativity was narrowly defined in this study using five representative items, utilizing existing large-scale panel data, a national data, obtained from the Korean Educational Development Institute (KEDI). Future researchers should pay attention to this when dealing with abstract constructs like creativity using only five items. Also, missingness is not completely random (there exists a weak correlation between missingness and relationship with parents in year 5). Although the effect of missingness is almost ignorable, future researchers may impute missing data from time point 3 to time point 5, treating the missing data as missing at random. Finally, we used students’ self-report as a measure of creativity. Although using self-report questionnaires is one of the four major approaches to creativity measure (Long et al., 2022), it has limitations in that self-report may include individuals’ subjective judgment (Biernat, 2003) and be related to reliability issues when reporting one’s own attitudes and behaviors. We expect to have a more reliable measure of creativity and the influence of cognitive and social factors by combining a self-report method together with an existing creativity test that can complement constructs the self-report did not cover.

Despite this limitation, this study explored the nature of the Korean students’ creativity over 5 years during the critical periods in developing abstract thinking; further, it investigated how individual characteristics and environmental or social aspects interact in an academic performance-oriented Korean educational setting.

## Data availability statement

Publicly available datasets were analyzed in this study. This data can be found at: https://www.kedi.re.kr/khome/main/research/requestResearchData.do.

## Ethics statement

The study involving human participants was reviewed and approved by the Research Board in Korean Educational Development Institute (IRB number: 2014-32-09-09-N, 2016-25-07-N). The data in this study were collected based on the written informed consent provided by patients/participants.

## Author contributions

H-sP and SeK: methodology and writing—original draft preparation. Hs-P: software, resources, data curation, and formal analysis. SeK and SuK: validation, and writing—review and editing. SuK: investigation and supervision. H-sP and SuK: investigation and supervision. SuK: project administration and funding acquisition. H-sP, SeK, and SuK: conceptualization. All authors have read and agreed to the published version of the manuscript.

## Funding

This work was supported by the Incheon National University Research Grant in 2021 and the National Research Foundation of Korea (NRF) grant funded by the Korea government (MSIT) (No. 2022R1A2C1010310).

## Conflict of interest

The authors declare that the research was conducted in the absence of any commercial or financial relationships that could be construed as a potential conflict of interest.

## Publisher’s note

All claims expressed in this article are solely those of the authors and do not necessarily represent those of their affiliated organizations, or those of the publisher, the editors and the reviewers. Any product that may be evaluated in this article, or claim that may be made by its manufacturer, is not guaranteed or endorsed by the publisher.
